# Highly different effects of phage therapy and antibiotic therapy on immunological responses of chickens infected with *Salmonella enterica* serovar Typhimurium

**DOI:** 10.3389/fimmu.2022.956833

**Published:** 2022-09-23

**Authors:** Łukasz Grabowski, Grzegorz Węgrzyn, Alicja Węgrzyn, Magdalena Podlacha

**Affiliations:** ^1^ Laboratory of Phage Therapy, Institute of Biochemistry and Biophysics, Polish Academy of Sciences, Gdansk, Poland; ^2^ Department of Molecular Biology, Faculty of Biology, University of Gdansk, Gdansk, Poland

**Keywords:** phage therapy, *Salmonella enterica* infection, chicken immunity, stress hormones, cytokines, safety and efficacy

## Abstract

The appearance of bacteria resistant to most or even all known antibiotics has become a serious medical problem. One such promising and effective alternative form of therapy may be the use of phages, the administration of which is considered to be safe and highly effective, especially in animals with drug-resistant infections. Although there have been no reports to date suggesting that bacteriophages can cause any severe complications or adverse effects, we still know little about their interactions with animal organisms, especially in the context of the functioning of the immune system. Therefore, the aim of the present study was to compare the impact of the application of selected bacteriophages and antibiotics (enrofloxacin and colistin), commonly used in veterinary medicine, on immune functions in *Salmonella enterica* serovar Typhimurium-infected chickens. The birds were infected with S. Typhimurium and then treated with a phage cocktail (14 days), enrofloxacin (5 days), or colistin (5 days). The concentrations of a panel of pro-inflammatory cytokines (IL-1β, IL-6, IFN-γ, IL-8, and IL-12) and cytokines that reveal anti-inflammatory effects (IL-10 and IL-4), the percentage of lymphocytes, and the level of stress hormones (corticosterone and cortisol), which significantly modulate the immune responses, were determined in different variants of the experiment. The phage cocktail revealed anti-inflammatory effects when administered either 1 day after infection or 2 days after *S*. Typhimurium detection in feces, as measured by inhibition of the increase in levels of inflammatory response markers (IL-1β, IL-6, IFN-γ, IL-8, and IL-12). This was also confirmed by increased levels of cytokines that exert an anti-inflammatory action (IL-10 and IL-4) following phage therapy. Moreover, phages did not cause a negative effect on the number and activity of lymphocytes’ subpopulations crucial for normal immune system function. These results indicate for the first time that phage therapy not only is effective but also can be used in veterinary medicine without disturbing immune homeostasis, expressed as cytokine imbalance, disturbed percentage of key immune cell subpopulations, and stress axis hyperactivity, which were observed in our experiments as adverse effects accompanying the antibiotic therapy.

## Introduction

The systematically increasing number of antibiotic-resistant bacteria is a global problem, which is the greatest challenge of modern medicine (1). Without the development of alternative therapeutic strategies, there is a serious risk that the situation will be similar to that before the invention of antibiotics (2). The importance of the problem is underscored by the fact that all major public health organizations have identified the fight against microbial drug resistance as a priority action (3). The search for new drugs against bacterial infections, however, is severely hampered for several reasons. These include the high cost of ongoing research, the long duration, and the relatively high risk of rapid development of resistance (4). For these reasons, bacteriophage therapy, or phage therapy for short, has emerged as an alternative in the fight against bacterial infections (5).

In addition to being widespread in nature, bacteriophages are also found in foods, as well as in human and animal bodies. After oral administration of bacteriophage, they quickly enter the bloodstream and can penetrate various organs (liver, spleen, or kidneys) *via* the lymphatic vessels and are then excreted in the urine. It is also important that they show the ability to penetrate the blood–brain barrier, retaining their antibacterial potential (6). The ability of lytic phages to kill bacterial cells makes them potential candidates for use not only in combating bacterial infections but also in preventing infectious diseases. The potent bactericidal activity of phages against both Gram-positive and Gram-negative bacteria was confirmed *in vitro* and *in vivo* (7). A unique feature of phages is their ability to replicate in bacterial cells, allowing more virions to be obtained directly at the site of infection. This, in turn, means that even a single administration of phage can have greater efficacy than multiple administrations of antibiotics (8). Of particular note is the fact that phages are able to kill bacteria resistant to commonly used antibiotics; this effect is already observed with a single dose (9). Despite the fact that research on the potential use of phages in clinical practice dates back to the 1920s, knowledge about the effects of phage therapy on the functioning of the human body is still incomplete, especially when it comes to the immune system (10).

In our recently published study, we compared the effects of phage therapy (a cocktail of bacteriophages vB_SenM-2 and vB_Sen-TO17 (11–13)) to the therapy with antibiotics (enrofloxacin and colistin) in *Salmonella enterica* serovar Typhimurium-infected chickens. It was demonstrated that the efficacies of both tested therapies were high when the application of the therapeutic agent (either phage cocktail or antibiotic) was started 1 day after the bacterial infection, as evidenced by the elimination of *S.* Typhimurium from the gastrointestinal tract (GIT) just next day after the onset of treatment. Later initiation of phage treatment was considerably less effective, as *S.* Typhimurium could be detected in feces and cloaca swabs for the next 2–4 days. Importantly, we were not able to observe the development of phage and antibiotic resistance of *S.* Typhimurium throughout the experiment. While antibiotics caused considerable changes in the GIT microbiome, such changes in phage-treated chickens were only transient (14). These results indicated that phage therapy can be as effective as the use of antibiotics in the eradication of *S.* Typhimurium from GIT of chickens; however, the immune responses of the birds to both methods of treatment were not tested.

Therefore, the aim of the present study was to determine the chicken immune response to phage therapy by examining the concentrations of cytokines, the percentage of lymphocytes, and the levels of stress hormones. The experimental material came from the large-scale experiment described previously (14). The immunological response to phage therapy was compared to the response to treatment with antibiotics (enrofloxacin and colistin) whose adverse effects are being increasingly reported in the literature (15, 16).

## Materials and methods

### Animals

The experiments were performed with chickens (*Gallus gallus domesticus*) that were delivered by a breeder (registration number PL28036602, Poland) and were non-genetically modified organism (non-GMO).

During the experiments, the chickens were located in the Experimental Infection Pavilion at the Department of Bird Diseases, Faculty of Veterinary Medicine, University of Warmia and Mazury in Olsztyn, Poland. This building was equipped with a high-efficiency particulate absorbing (HEPA) filter system and automation to maintain a cascade of pressures in the sanitary corridors, boxes, and locks, designed to exclude the possibility of contamination of the experimental rooms.

The animals were housed in 8 m^2^ boxes, 25 chickens in each, with an average humidity of 75%, under conditions of regular light–dark cycles (12-h day/12-h night, at light intensity 10 lx) and forced ventilation (17 air changes per hour). The temperature was reduced from 33°C (beginning of the experiment) to 22°C (end of the experiment). Chickens were fed complete forage in the *ad libitum* system. Water was available around the clock.

All experiments were approved by the Local Ethics Committee for Experimental Animals in Olsztyn (permission number 62/2019 dated 30 July 2019).

### Bacteriophages and bacterial strain

Bacteriophages vB_Sen-TO17 and vB_SenM-2 came from the Collection of the Department of Molecular Biology, University of Gdansk. They were characterized in previous works (11, 12), and their safety was confirmed in *in vitro* experiments with the chicken fibroblast model (UMNSAH/DF-1) and in *in vivo* studies with the *Galleria mellonella* animal model (13).

*S. enterica* serovar Typhimurium (strain KOS 13) was obtained from the National *Salmonella* Centre at the Medical University of Gdańsk (Poland), and *S. enterica* serovar Heidelberg came from the Collection of the Department of Molecular Biology, University of Gdansk.

Isolation of *S*. Typhimurium in chicken fecal samples and cloacal swabs was conducted in accordance with ISO 6579-1:2017 standards. The serotype of the isolated bacteria was confirmed by serological identification using the SIT EnTy Kit from Immunolab (Gdansk, Poland).

Chicken fecal sample or cloacal swab measuring 0.5 g was mixed with 5 ml of peptone water and incubated for 30 min at 37°C. After the incubation, serial dilutions in peptone water were prepared, and 50 µl of each dilution was spread onto plates with CHROMagar Salmonella PLUS medium. The plates were incubated at 37°C overnight. Purple *S*. Typhimurium colonies were then counted to calculate CFU/ml in either fecal samples or cloacal swabs.

### The preparation of phage cocktail

Phage lysates were prepared according to the protocol described by Kosznik-Kwaśnicka et al. (13). Overnight culture of *S. enterica* was added to fresh LB medium (Bio-Shop, Burlington, Canada) at a ratio (v/v) of 1:100 and then cultured with shaking at 37°C until OD_600_ = 0.15 (1.5 × 10^8^ CFU/ml).

The bacteria were then infected with the appropriate bacteriophage at the multiplicity of infection (m.o.i.) of 0.5. Then obtained mixture was cultured with shaking at 150 r.p.m. at 37°C until lysis was completed. To purify the phage lysate, polyethylene glycol 8000 (PEG8000) (BioShop, Burlington, ON, Canada) was added to a final concentration of 10% and mixed with a magnetic stirrer (Carl Roth, Karlsruhe, Germany) overnight at 4°C. The lysate was then centrifuged at 10,000 × *g* for 30 min, at 4°C (Avanti JXN-26, rotor JLA-8000, Beckman Coulter, Indianapolis, IN, USA) to obtain a pellet, which was then suspended in 0.89% NaCl (Alchem, Torun, Poland). To remove PEG8000, the lysate was treated with 2 ml of chloroform and centrifuged at 4,000 × *g* for 15 min, at 4°C (Avanti JXN-26, rotor JS-13.1, Beckman Coulter, Indianapolis, IN, USA). The procedure was repeated until PEG8000 was not observed. Obtained lysates were then ultracentrifuged in a sucrose gradient (Sigma Aldrich, Saint Louis, MO, USA) at 95,000 × *g* (Optima XPN-100, rotor SW32.1 Ti, Beckman Coulter, Indianapolis, IN, USA) for 2.5 h at 10°C. To remove residual sucrose, lysates were dialyzed against 0.89% NaCl for 7 days at 4°C. The levels of bacterial endotoxin were tested using Purified Thermo Scientific™ Pierce™LAL Chromogenic Endotoxin Quantitation Kit (no. 12117850, Thermo Fisher Scientific Inc., Paisley, UK) in accordance with the previously published protocol (13). Thus, purified and tested lysates of bacteriophage vB_SenM-2 and vB_Sen-TO17 were combined in a 1:1 ratio (1 × 10^9^ PFU/ml of each phage). The cocktail was suspended in 20 mM of CaCO_3_ before administration to the animals.

### The evaluation of phage therapy effects in the chicken model

**Figure 1:**
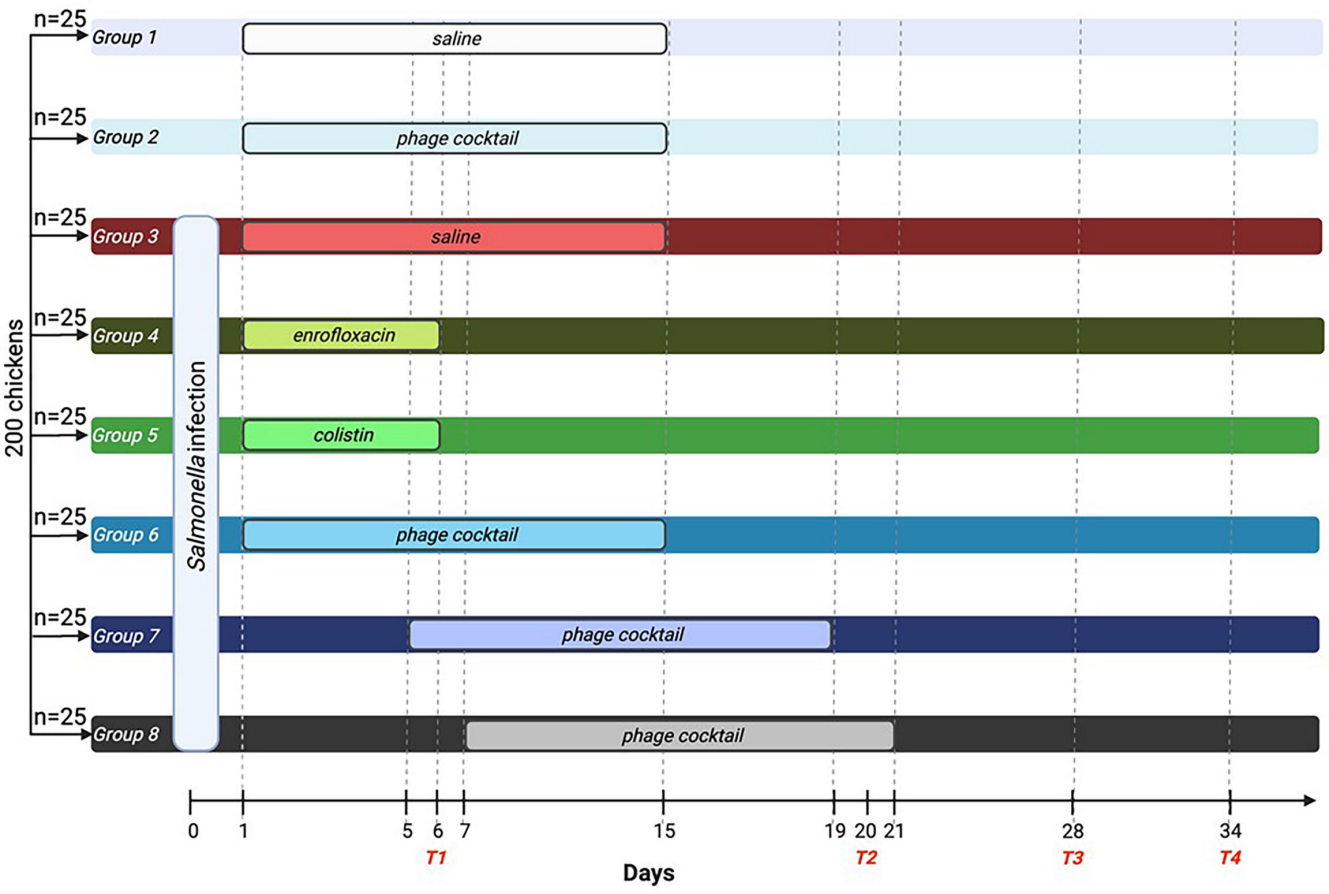
Diagram of the conducted experiment. A total of 200 birds were randomly divided into eight groups with 25 chickens each. On day 0 of the experiment, groups 3–8 were infected with *Salmonella* Typhimurium. Groups 1 and 2 were not infected with the bacteria. Groups 4 (enrofloxacin) and 5 (colistin) were treated with antibiotics for 5 days. Groups 6–8 were treated with phage cocktail for 14 days. However, group 6 received the cocktail 1 day after infection, while group 7 received the cocktail 2 days after *Salmonella* detection in the feces, and group 8 received the cocktail 4 days after bacteria detection in the feces. On days 6, 20, 28, and 34, blood was drawn, and the animals were sacrificed. T1, termination 1; T2, termination 2; T3, termination 3; T4, termination 4.

Two hundred 7-day-old chickens were randomly divided into eight groups, each with 25 chickens. Group 1 received saline, and group 2 received a phage cocktail from day 1 to day 15 of the experiment, and these chickens were not infected. In group 1, the effect of the administration procedure on the parameters studied was tested, while in group 2, the immune response after phage cocktail administration was tested. Group 3 was the positive control; chickens were infected and then treated with 0.89% NaCl until the 15th day of the experiment. On day 0 of the experiment, groups 3–8 were infected by administering 1 ml of *S*. Typhimurium (10^6^ CFU/ml) suspended in 0.89% NaCl into the beak. Twenty-four hours after infection (day 1 of the experiment), chickens were treated with enrofloxacin [Scanflox, Scanvet, Warsaw, Poland; dose 10 mg/kg per day (group 4)] and colistin [Colisol, Ceva Animal Health, Warsaw, Poland; dose 120.000 IU/kg per day (group 5)] for 5 days or phage cocktail (group 6 received 1 ml of phage cocktail for 14 days). Groups 4 and 5 were designed to evaluate the immune response of chickens after antibiotic treatment. Group 7 received a phage cocktail 2 days after the detection of *S*. Typhimurium in feces, while group 8 received a phage cocktail 4 days after the detection of bacteria in feces. Both of these groups received the phage cocktail for 14 days. Groups 6, 7, and 8 were designed to evaluate the substitution of immune parameters depending on the timing of phage cocktail initiation. On day 6 of the experiment, after the end of antibiotic treatment, 5 ml of blood was collected from five chickens of each group for further experiments, and the animals were then sacrificed in a CO_2_ chamber (termination 1). The organs of the animals were further analyzed. On day 20 of the experiment, following the completion of phage therapy in group 6, blood was collected from another five chickens (in groups 1–6) and 10 chickens (in groups 7 and 8), and the animals were then sacrificed (termination 2). Subsequent blood sampling and sacrifice were performed on day 28 of the experiment (five chickens from each group; termination 3) and day 34 of the experiment (10 chickens from each group; termination 4). A graphical diagram of the experiment is shown in **Figure 1**.

### Blood collection

Blood samples of 5 ml were collected from each chicken before sacrifice. Animals were immobilized by gentle restraint, and blood was harvested using heparinized syringes tipped with a 25-gauge, 1-in-long needle inserted into the brachial wing vein at a shallow angle (approximately 10°–20°) into tubes containing sodium heparin. Each blood sample collected was immediately divided according to the course of further determination: 1 ml of whole blood was used to obtain the results of flow cytometry, while the remaining blood was centrifuged (1,800 × *g* for 15 min at 4°C) to obtain plasma, which was subjected to deep freezing (−80°C) until further analysis.

### Determination of the percentage of lymphocyte populations (T, B) and subpopulations of T 171 helper (Th, TCD4+) and T cytotoxic (Tc, TCD8+) in peripheral blood by flow cytometry

Cytometric analysis of the lymphocyte population was performed after centrifugation of blood in a Ficoll gradient (1,113 × *g*, 30 min, 4°C) and uropolin according to the procedure described previously (17). Peripheral blood mononuclear cells (PBMCs; mainly lymphocytes, and monocytes), isolated by this method, were suspended at a concentration of 10^7^ cells/ml. Twenty-five microliters of the PBMC suspension was added to 25 µl of AntiChicken antibodies for B lymphocytes (Bu-1-FITC, AV20 cat. no. 8395-02, SouthernBiotech), for T lymphocytes, and their subpopulations (CD4: CD4-PE clone CT-4, cat. no. 8210-09, SouthernBiotech; CD8: CD8a-FITC clone 3-298, cat. no. 8405-02, SouthernBiotech). The samples were protected from light and incubated for 20 min in the dark at room temperature. After incubation, 700 µl of phosphate-buffered saline (PBS) and 25 µl of fixative solution (Fixative Solution IOTest O3, Beckman Coulter, Brea, CA, USA) were added to them. The percentage of lymphocyte populations and subpopulations was determined by flow cytometry using the FACSVerse cytometer (Becton Dickinson) and BD FACSuite software version 1.0.5. The separation into subpopulations was based on the surface expression of CD4 (helper T cells, Th, TCD4+) or CD8 (cytotoxic T cells, Tc, TCD8+). The total number of lymphocytes and their subpopulations was calculated based on the total number of leukocytes and the percentage of T, B TCD4+, and TCD8+ lymphocytes.

### Determination of pro-inflammatory cytokines (IL-1β, IL-6, IFN-γ, IL-8, and IL-12), cytokines that reveal anti-inflammatory effects (IL-10 and IL-4), and corticosterone and cortisol concentrations (ELISA) in peripheral blood plasma

A sandwich immunoenzymatic ELISA test, which involves the formation of immune complexes between an antigen and two layers of antibodies conjugated to a specific enzyme, was used to determine the concentration of the mentioned cytokines and hormones. The procedure was performed according to the instructions included with each set of commercial reagents (Wuhan Fine Biotech Co., Ltd., Wuhan, China).

All reagents and samples were brought to room temperature (20°C–25°C) before use. Into each well of the titration plate (96-well Nunc plate), coated with cytokine- or hormone-specific monoclonal antibodies, 100 µl (all cytokines) or 50 µl (corticosterone and cortisol) of buffer, test samples, or respective standards were added in duplicate. Plates were covered and incubated at 37°C for 90 min (all cytokines) or 45 min (corticosterone and cortisol). Then, the plate contents were drained and washed with prepared buffer three times to remove excess unbound antigens. Then, 100 µl (all cytokines) or 50 µl (corticosterone and cortisol) of a solution of specific polyclonal biotinylated antibody conjugated with the enzyme for the appropriate cytokine or hormone was added. The plates were coverslipped, incubated at 37°C for 60 min, then drained again, and washed three times. Subsequently, 100 µl of streptavidin-labeled horseradish peroxidase enzyme solution was added and incubated for 30 min at 37°C. The plates were then drained and washed five times, and 90 µl of 3,3′,5,5′-tetramethylbenzidine (a colored substrate for horseradish peroxidase) solution was added and incubated for 30 min in the dark at 37°C. The reaction was stopped by adding 50 µl of blocking solution, which changed the color of the product (from blue to yellow). Absorbance was measured at 10 min after stopping the reaction using a Multiskan FC microplate reader (Thermo Fisher Scientific, Waltham, MA, USA), coupled with Skanlt 6.1.1. RE software, which analyzes spectrophotometric color intensity, plots a standard curve based on the standards used, and reads the concentration values of the individual cytokines or hormones in the plasma samples tested. The results obtained were given in pg/ml or ng/ml. The minimum sensitivity of the test was 15.625 pg/ml for IL-8, IL-12, IL-10, and IL-4; 31.25 pg/ml for IL-1β, IFN-γ, and IL-6; and 2.81 ng/ml for corticosterone and cortisol.

### Statistical analysis

The results are presented as mean ± standard deviation (SD). For statistical analysis of the results, SPSS 21.0 (SPSS Inc., Armonk, NY, USA) software was used. The normality of the distribution of variables was checked with the Kolmogorov–Smirnov test and the homogeneity of the variances with Levene’s test. If the assumptions of normality of distribution and/or homogeneity of variance were not met, the Kruskal–Wallis test and post-hoc Dunn test were applied. Once both assumptions were met, the analysis was carried out on the basis of ANOVA and post-hoc Tukey’s test. A *p*-value lower than 0.05 was considered statistically significant.

## Results

### Changes in plasma levels of pro-inflammatory cytokines and cytokines that reveal anti-inflammatory effects in chickens subjected to phage therapy and antibiotic treatment

The experiments with chickens infected with *S.* Typhimurium and treated with either the phage cocktail or antibiotic were performed as depicted in **Figure 1**.

**Figure 2:**
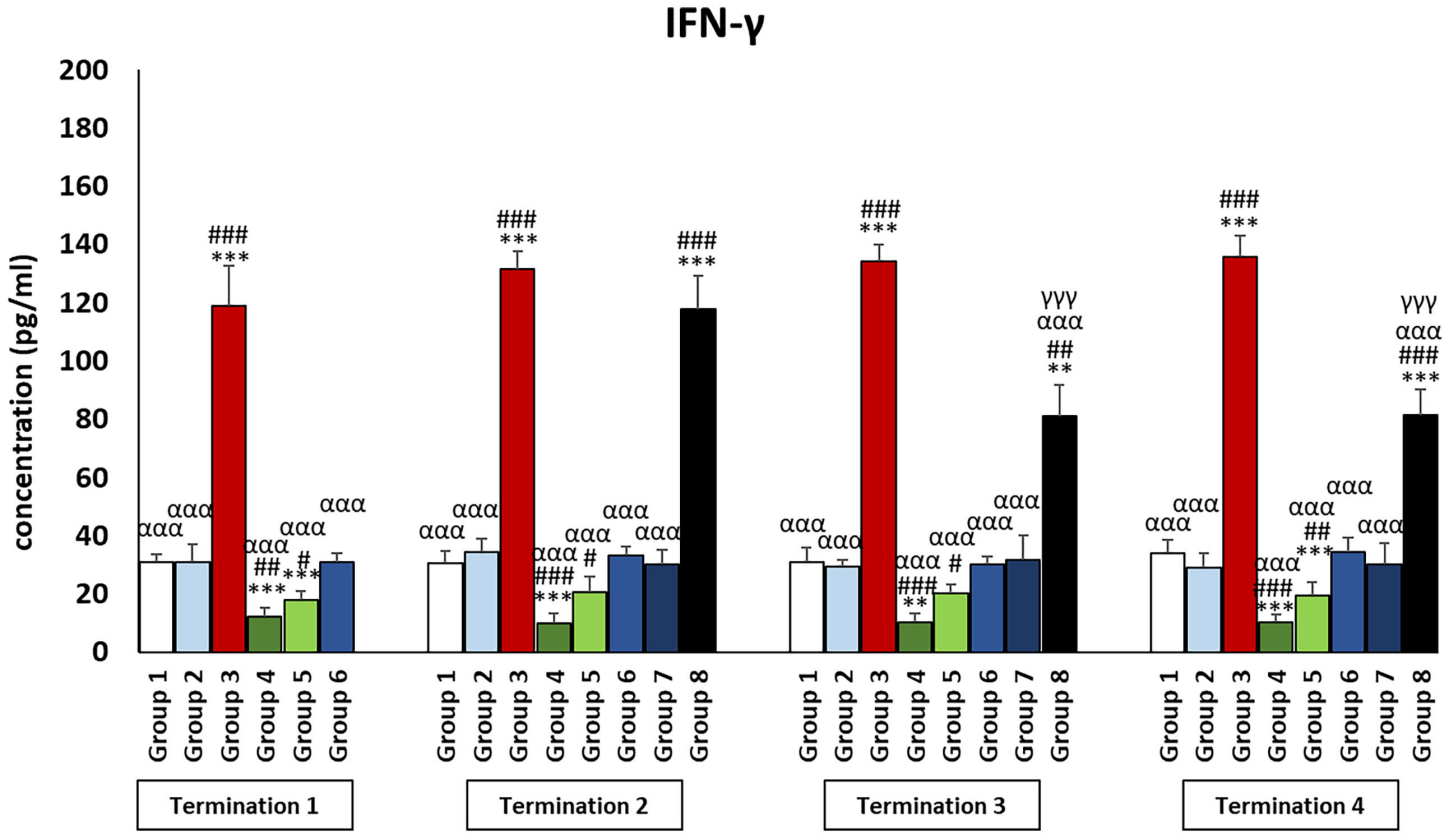
Changes in the levels of IFN-γ in the blood of chickens receiving phage therapy or antibiotic therapy after 6, 20, 28, and 34 days of experiments. Results are presented as mean values ± SD. Statistical analyses were performed by Kruskal–Wallis test and post-hoc Dunn test or ANOVA and post-hoc Tukey’s test. The significance of differences between controls and particular treated groups is observed and marked by the following: asterisks (*) vs. saline control; (#) vs. phage control (group 2); (a) vs. infected control (group 3); (g) vs. termination 2. p < 0.001 (***, ###, ααα, γγγ), p < 0.01 (**, ##), and p < 0.05 (*, #).

**Figure 3:**
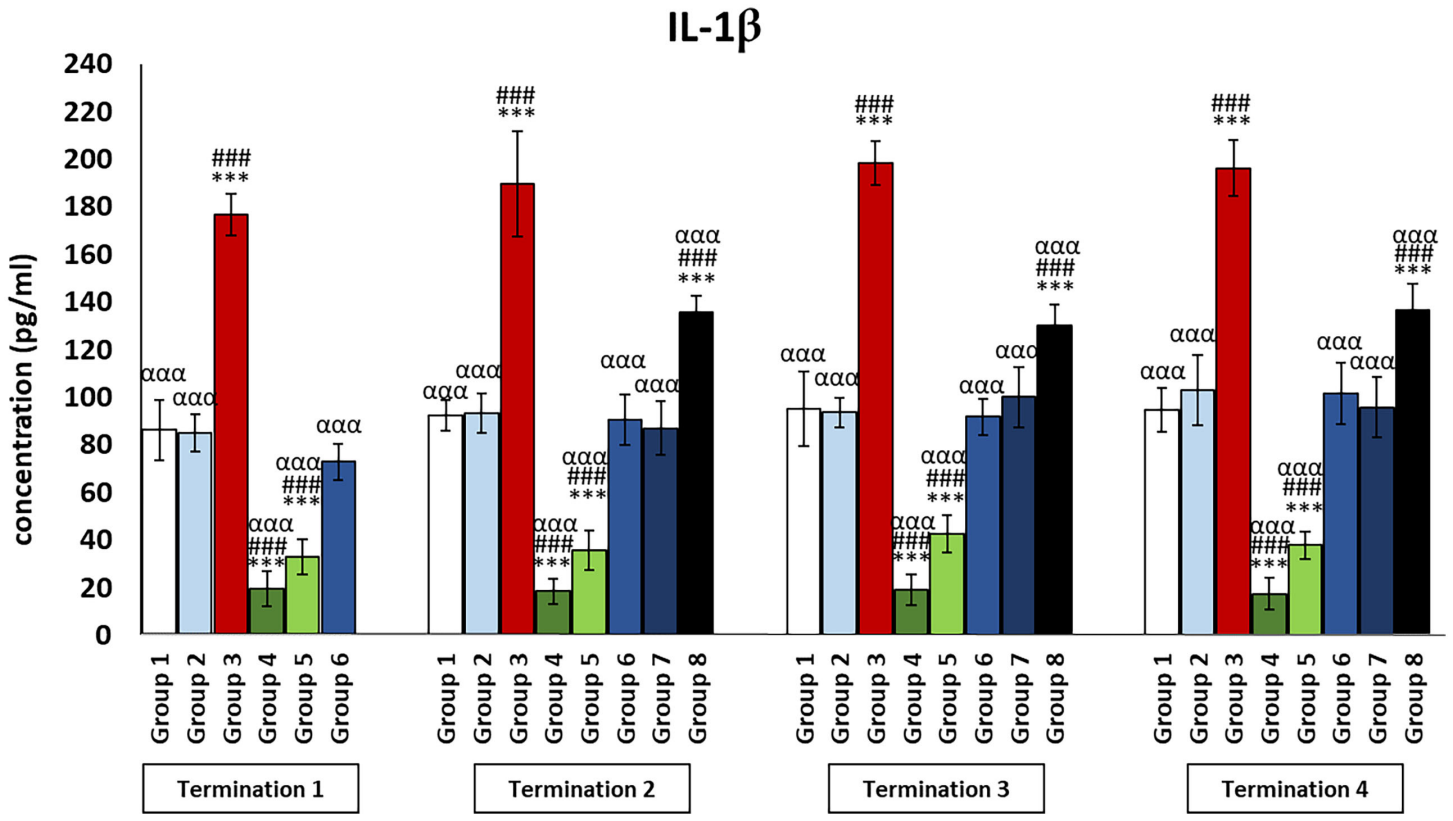
Changes in the levels of IL-1β in the blood of chickens receiving phage therapy or antibiotic therapy after 6, 20, 28, and 34 days of experiments. Results are presented as mean values ± SD. Statistical analyses were performed by Kruskal–Wallis test and post-hoc Dunn test or ANOVA and post-hoc Tukey’s test. The significance of differences between controls and particular treated groups is observed and marked by the following: asterisks (*) vs. saline control; (#) vs. phage control (group 2); (a) vs. infected control (group 3); (g) vs. termination 2. p < 0.001 (***, ###, ααα).

It was observed that the levels of all pro-inflammatory interleukins were significantly increased in the group that received the phage cocktail latest, i.e., 4 days after detection of *Salmonella* in the feces (group 8) as compared to the uninfected control groups (groups 1 and 2) (*p* ≤ 0.001). Moreover, the level of IFN-γ (**Figure 2**) in this group was the highest on day 20 of the experiment (termination 2), was not significantly different from that in the infected control group (group 3), and then decreased significantly as compared to the uninfected control groups (groups 1 and 2) on day 28 (termination 3, *p* ≤ 0.01) and day 34 of the experiment (termination 4, *p* ≤ 0.001). Treatment with enrofloxacin (group 4) and colistin (group 5) resulted in a significant decrease in the levels of all tested pro-inflammatory cytokines (**Figures 2**, **3**; **Supplementary Figures 1**–**3**) compared to all control groups (groups 1, 2, and 3) and a decrease throughout the experimental period (*p* ≤ 0.001). IL-8 levels (**Supplementary Figure 2**) in group 6 were significantly increased on day 20 (termination 2, *p* ≤ 0.01) and day 28 of the experiment (termination 3, *p* ≤ 0.01) compared to the phage-treated control group (group 2). Analogous results were obtained for chickens treated with a phage cocktail 2 days after the detection of *Salmonella* in feces (group 7).

**Figure 4:**
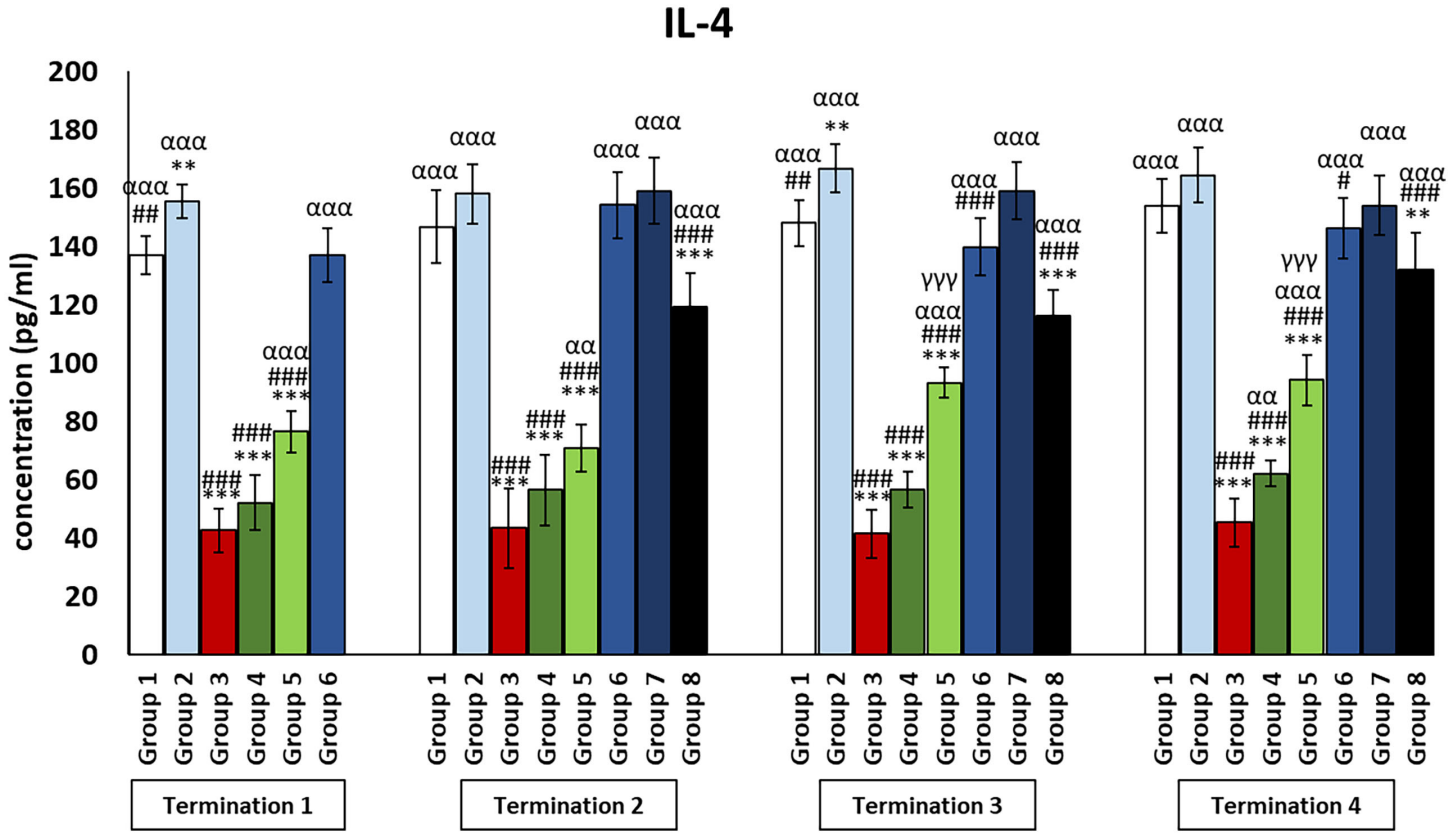
Changes in the levels of IL-4 in the blood of chickens receiving phage therapy or antibiotic therapy after 6, 20, 28, and 34 days of experiments. Results are presented as mean values ± SD. Statistical analyses were performed by Kruskal–Wallis test and post-hoc Dunn test or ANOVA and post-hoc Tukey’s test. The significance of differences between controls and particular treated groups is observed and marked by the following: asterisks (*) vs. saline control; (#) vs. phage control (group 2); (a) vs. infected control (group 3); (g) vs. termination 2. p < 0.001 (***, ###, ααα, γγγ), p < 0.01 (**, ##, αα), and p < 0.05 (#).

**Figure 5:**
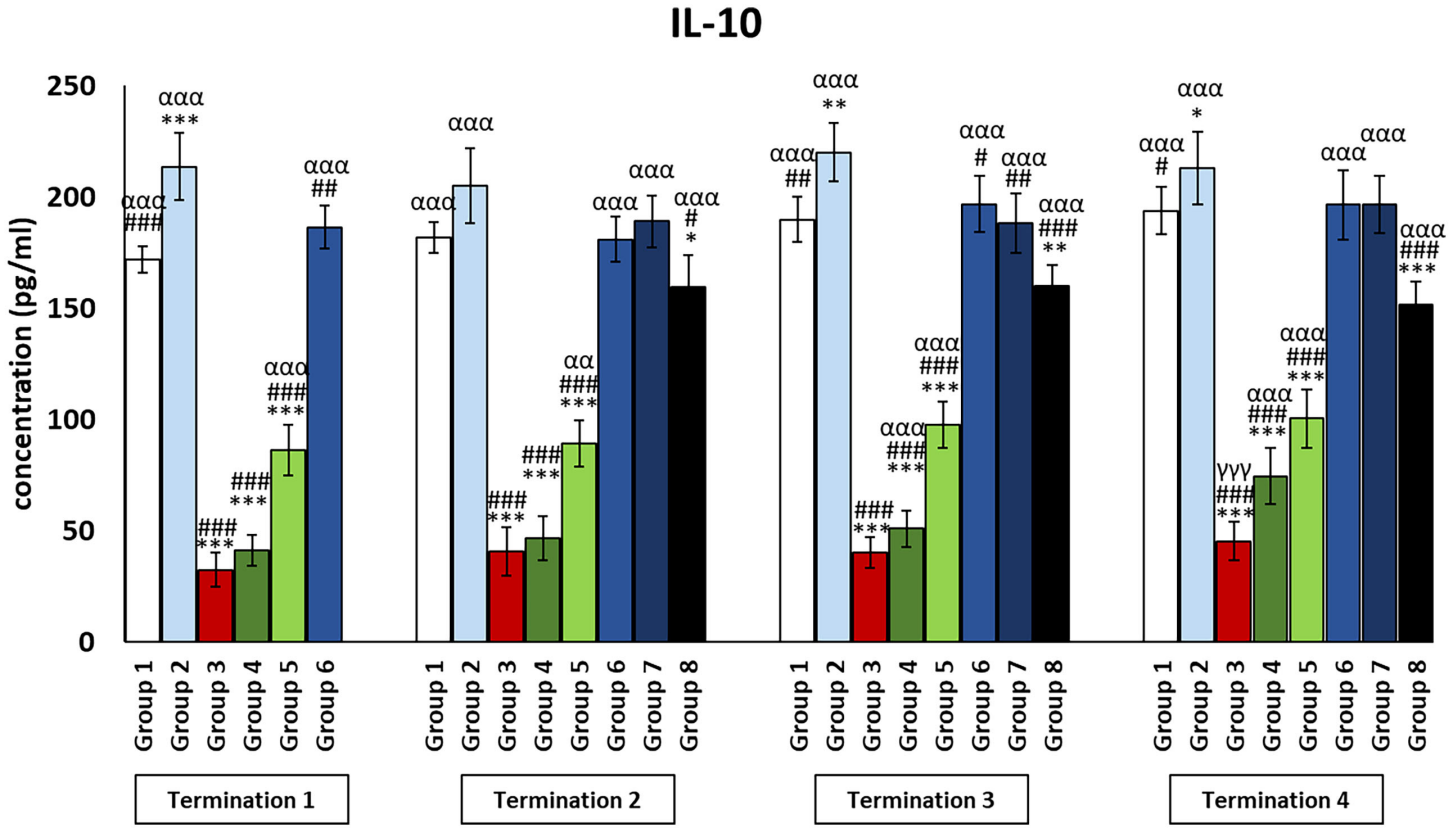
Changes in the levels of IL-10 in the blood of chickens receiving phage therapy or antibiotic therapy after 6, 20, 28, and 34 days of experiments. Results are presented as mean values ± SD. Statistical analyses were performed by Kruskal–Wallis test and post-hoc Dunn test or ANOVA and post-hoc Tukey’s test. The significance of differences between controls and particular treated groups is observed and marked by the following: asterisks (*) vs. saline control; (#) vs. phage control (group 2); (a) vs. infected control (group 3); (g) vs. termination 2. p < 0.001 (***, ###, ααα, γγγ), p < 0.01 (**, ##, αα), and p < 0.05 (*, #).

Interestingly, for the control group receiving the phage cocktail (group 2), the levels of cytokines that exert anti-inflammatory actions (**Figures 4**, **5**) were significantly increased as compared to the saline control group (group 1). For the group receiving a phage cocktail 24 h after infection (group 6), interleukin levels were significantly changed as compared to the saline-treated control group (group 1). However, for the group that received the phage cocktail the latest (group 8), levels of cytokines that exert anti-inflammatory actions were significantly decreased as compared to the uninfected control groups (groups 1 and 2). For IL-4 (**Figure 4**), this was observed on day 6 (termination 1, *p* ≤ 0.01) and day 28 (termination 3, *p* ≤ 0.01) of the experiment, while the IL-10 level (**Figure 5**) was elevated on day 6 (termination 1, *p* ≤ 0.001), day 28 (termination 3, *p* ≤ 0.01), and day 34 (termination 4, *p* ≤ 0.05) of the experiment. The levels of interleukins that exert anti-inflammatory actions (**Figures 4**, **5**) in the antibiotic-treated groups (groups 4 and 5) were drastically reduced as compared to the uninfected control groups (groups 1 and 2) (*p* ≤ 0.001) throughout the experiment.

In the present experiments, due to the determination of many immune system parameters, a bacterial strain was chosen that does not generate increased chicken mortality. Therefore, in the course of the experiment, we do not observe an acute inflammatory reaction that would translate into a significant (100- or 1,000-fold) increase/decrease in the studied parameters. Nevertheless, it can be observed that due to the slower and gradual elimination of bacteria, bacteriophages maintain the cytokine balance in the organism at a level, comparable to the control group. In turn, as a result of antibiotic therapy, immune homeostasis can be disrupted, due to the rapid elimination of bacterial cells, resulting in a massive release of toxins that do not benefit the organism.

### Changes in the number of lymphocytes in the peripheral blood of chickens treated with phages and antibiotics

In the infected control group (group 3), T and Th CD4+ lymphocyte levels were dramatically increased throughout the experiment as compared to the uninfected control groups (groups 1 and 2) (*p* ≤ 0.001). Interestingly, in the group that was treated with the phage cocktail 1 day after infection (group 6), but also in the group in which phage therapy was started 2 days after the detection of bacteria in the feces (group 7), the levels of T and Th CD4+ lymphocytes were not statistically significantly different from those of the uninfected control groups (groups 1 and 2) throughout the experiment. However, we observed that in the group in which phage treatment was administered at the latest (group 8), the levels of T and Th CD4+ lymphocytes were dramatically increased and were not statistically significantly different from those of the infected control group (group 3).

In the case of the group in which phage treatment was administrated 1 day after infection (group 6), Tc CD8+ levels were not significantly different from the values obtained in the uninfected control groups (groups 1 and 2) on day 6 (termination 1), while on day 20 (termination 2, *p* ≤ 0.05) and on day 28 (termination 3, *p* ≤ 0.05), the levels were significantly reduced compared to groups 2 and 1, respectively. This trend continues on day 34 as well, except that the difference was in comparison to both control groups (group 1, *p* ≤ 0.001; group 2, *p* ≤ 0.01). A statistically significant decrease in the number of Tc CD8+ lymphocytes was observed in enrofloxacin-treated chickens (group 4) compared to uninfected controls (termination 1, groups 1 and 2 (*p* ≤ 0.05); termination 2, group 1 (*p* ≤ 0.05) and group 2 (*p* ≤ 0.01); termination 3, group 1 (*p* ≤ 0.001) and group 2 (*p* ≤ 0.01); and termination 4, groups 1 and 2 (*p* ≤ 0.001)).

**Figure 6:**
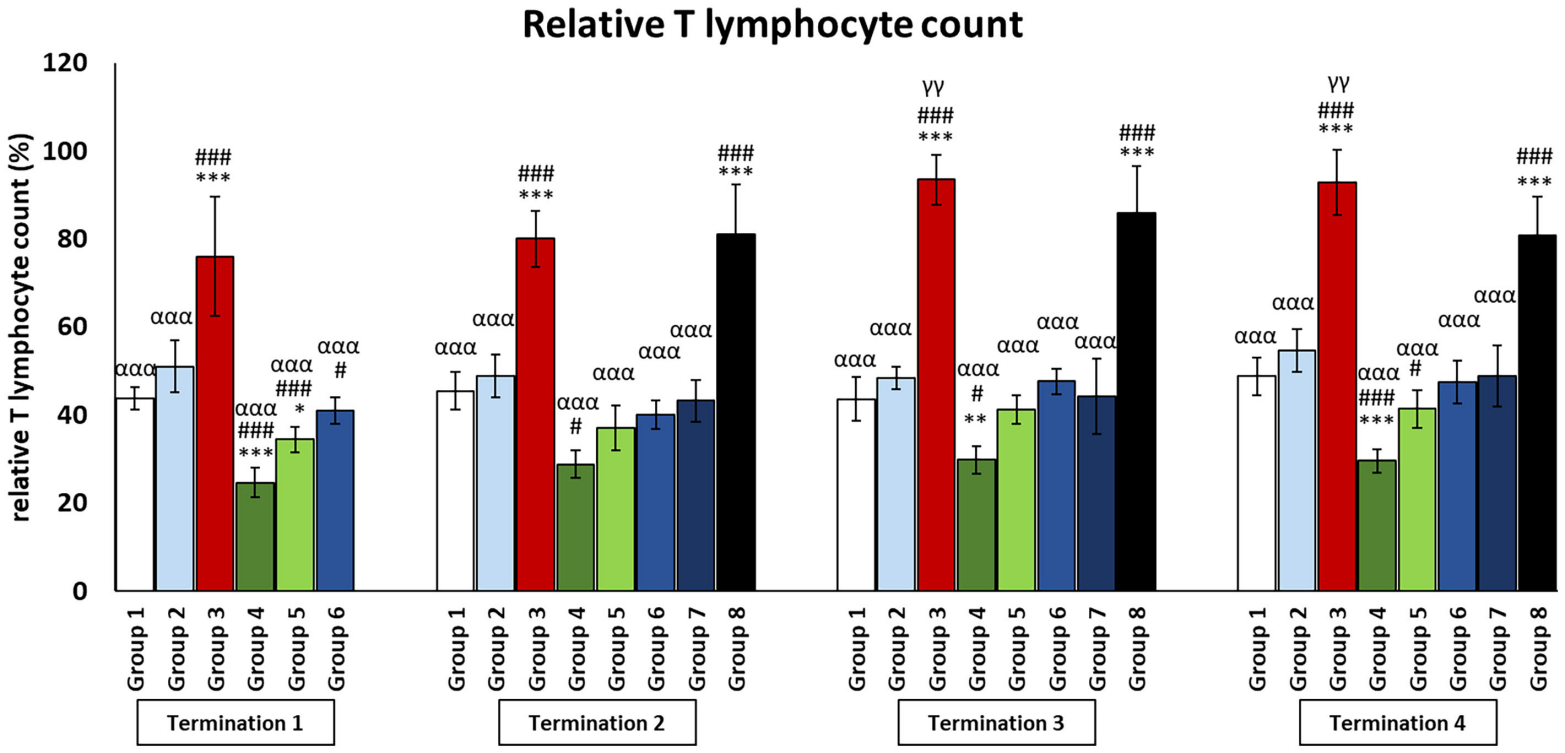
Changes in lymphocyte T counts in the blood of chickens receiving phage therapy or antibiotic therapy after 6, 20, 28, and 34 days of experiments. Results are presented as mean values ± SD. Statistical analyses were performed by Kruskal–Wallis test and post-hoc Dunn test or ANOVA and post-hoc Tukey’s test. The significance of differences between controls and particular treated groups is observed and marked by the following: asterisks (*) vs. saline control; (#) vs. phage control (group 2); (a) vs. infected control (group 3); (g) vs. termination 2. p < 0.001 (***, ###, ααα), p < 0.01 (**, γγ), and p < 0.05 (*, #).

The number of B lymphocytes in the infected control group (group 3) drastically increased as compared to the uninfected control groups (groups 1 and 2) throughout the experiment (**Figure 6**). It was also observed that the number of B lymphocytes in the group that received a phage cocktail 4 days after the detection of bacteria in feces (group 8) was increased throughout the experiment, relative to the control group receiving bacteriophages (group 2) (*p* ≤ 0.01). Interestingly, in the group treated with enrofloxacin (group 4), the number of B lymphocytes was not statistically significantly different from the uninfected control groups (groups 1 and 2) on day 6 of the experiment (termination 1), while it decreased significantly on day 20 (termination 2, *p* ≤ 0.05), day 28 (termination 3, *p* ≤ 0.01), and day 34 (termination 4, *p* ≤ 0.001) of the experiment compared to the saline-treated control group (group 1).

**Figure 7:**
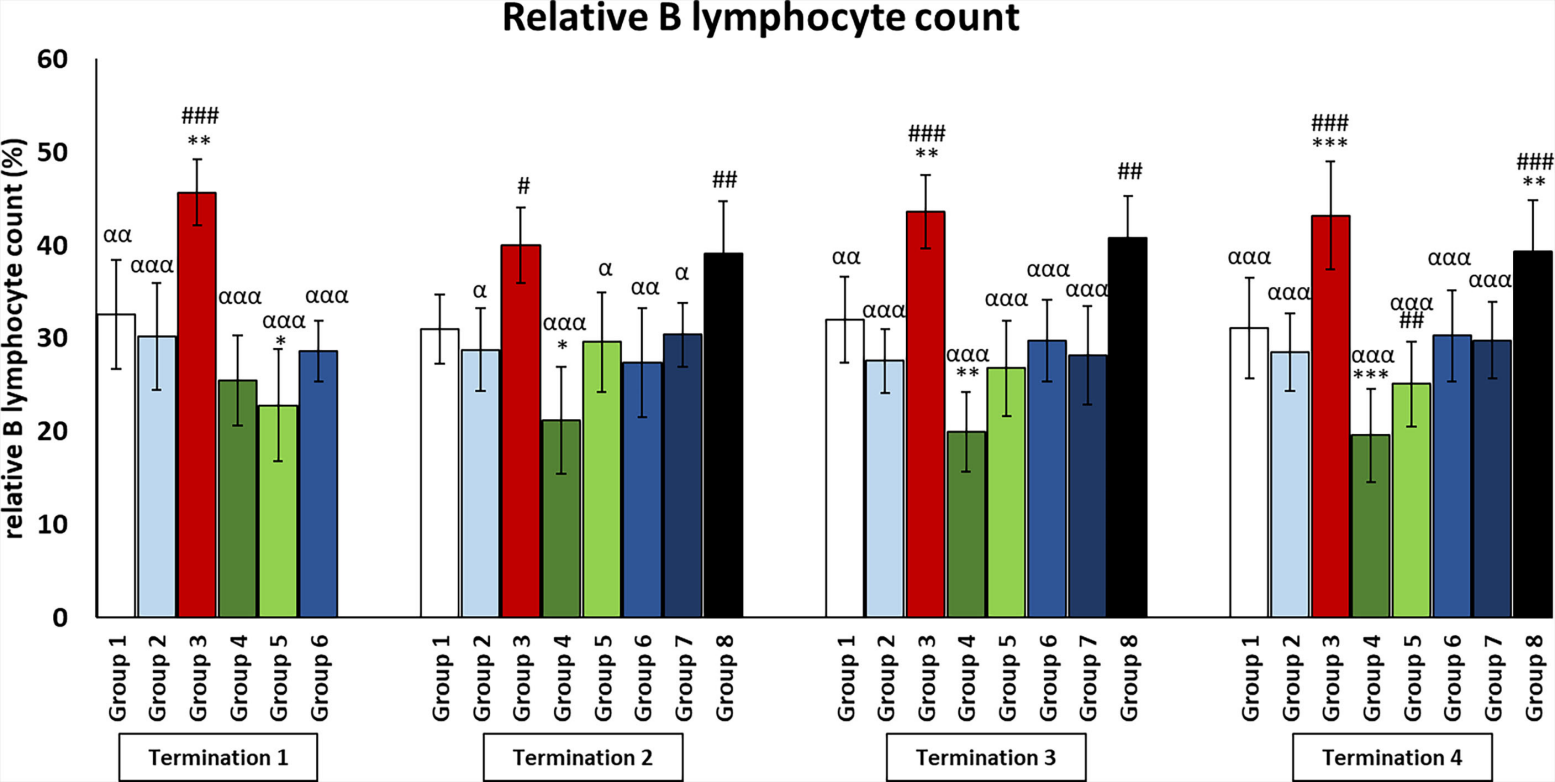
Changes in lymphocyte B counts in the blood of chickens receiving phage therapy or antibiotic therapy after 6, 20, 28, and 34 days of experiments. Results are presented as mean values ± SD. Statistical analyses were performed by Kruskal–Wallis test and post-hoc Dunn test or ANOVA and post-hoc Tukey’s test. The significance of differences between controls and particular treated groups is observed and marked by the following: asterisks (*) vs. saline control; (#) vs. phage control (group 2); (a) vs. infected control (group 3); (g) vs. termination 2. p < 0.001 (***, ###, ααα), p < 0.01 (**, ##, αα), and p < 0.05 (*, #, α).

As it is well known, the concentration of cytokines is one of the components of the defense system against various types of infections. Another important piece of this immune puzzle is the appropriate ratio of T and B lymphocytes, which determines the release of adequate amounts of the aforementioned mediators. The results show that the phage cocktail, as well as its supplementation to *S. enterica*-infected chickens, does not disrupt the physiological interaction between key cells of the immune system, unlike the antibiotics used (**Figures 6**, **7** and **Supplementary Figures 4**–**6**).

### Stress hormone levels in plasma of chickens subjected to phage therapy and antibiotic therapy

**Figure 8:**
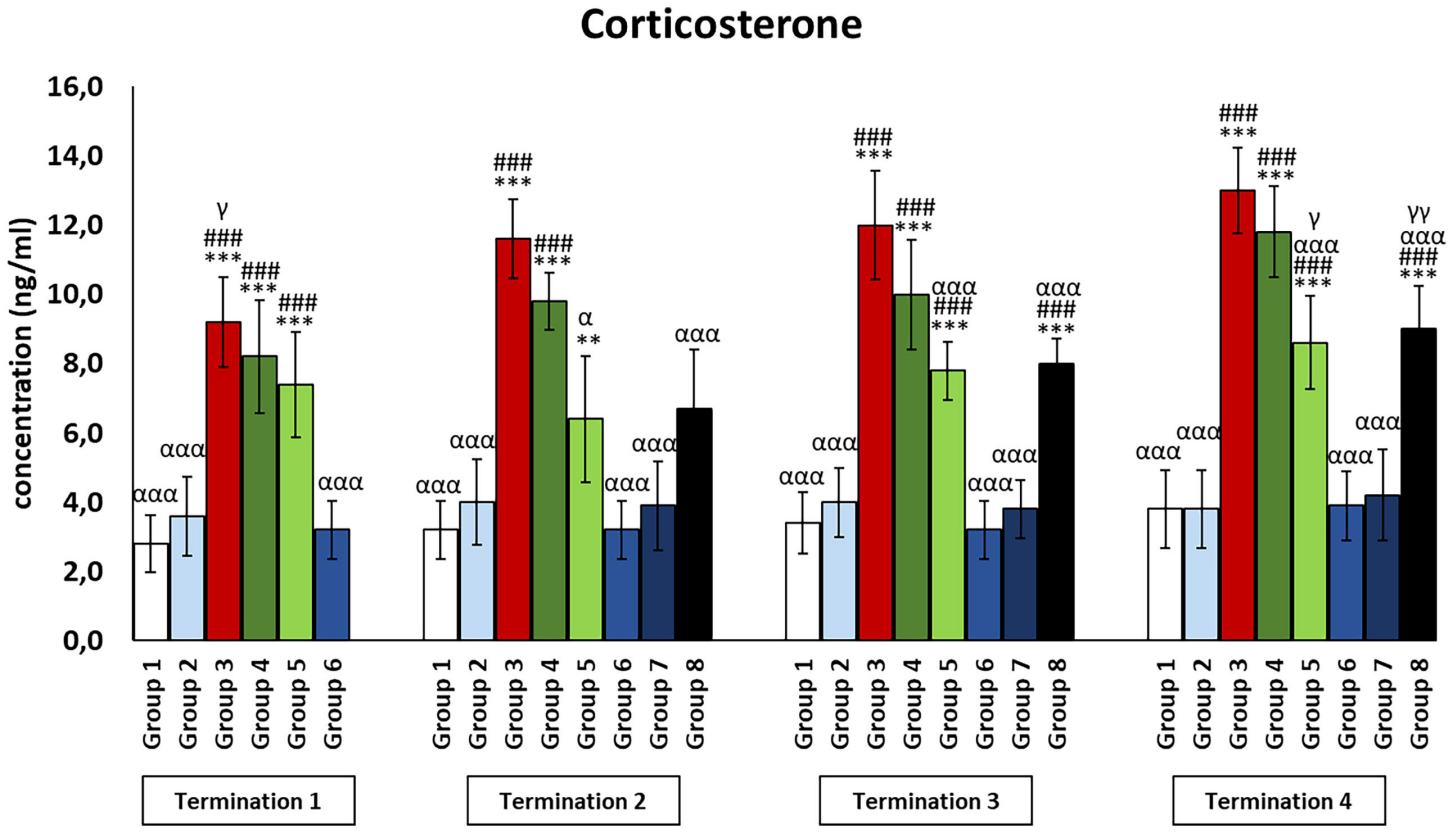
Changes in the levels of corticosterone in the blood of chickens receiving phage therapy or antibiotic therapy after 6, 20, 28, and 34 days of experiments. Results are presented as mean values ± SD. Statistical analyses were performed by Kruskal–Wallis test and post-hoc Dunn test or ANOVA and post-hoc Tukey’s test. The significance of differences between controls and particular treated groups is observed and marked by the following: asterisks (*) vs. saline control; (#) vs. phage control (group 2); (a) vs. infected control (group 3); (g) vs. termination 2. p < 0.001 (***, ###, ααα), p < 0.01 (**,γγ), and p < 0.05 (α, γ).

The infected control group (group 3) was observed to have significantly increased corticosterone levels as compared to the uninfected control groups (groups 1 and 2) (*p* ≤ 0.001) throughout the experiment (**Figure 8**). Moreover, corticosterone levels were also increased in the antibiotic-treated groups (groups 4 and 5). In the case of the enrofloxacin-treated group (group 4), the level was drastically increased as compared to the uninfected control groups (groups 1 and 2) throughout the experiment (*p* ≤ 0.001). In groups treated with colistin (group 5), corticosterone levels were elevated relative to the uninfected control groups (groups 1 and 2) on day 6 (termination 1, *p* ≤ 0.001), day 28 (termination 3, *p* ≤ 0.001), and day 34 (termination 4, *p* ≤ 0.001) of the experiment. Although the level of corticosterone in animals treated with the phage cocktail at the latest (group 8) was not significantly different from the uninfected control groups (groups 1 and 2) on day 20 of the experiment (termination 2), it was statistically significantly increased relative to values measured in these groups on day 28 (termination 3, *p* ≤ 0.001) and day 34 (termination 4, *p* ≤ 0.001).

**Figure 9:**
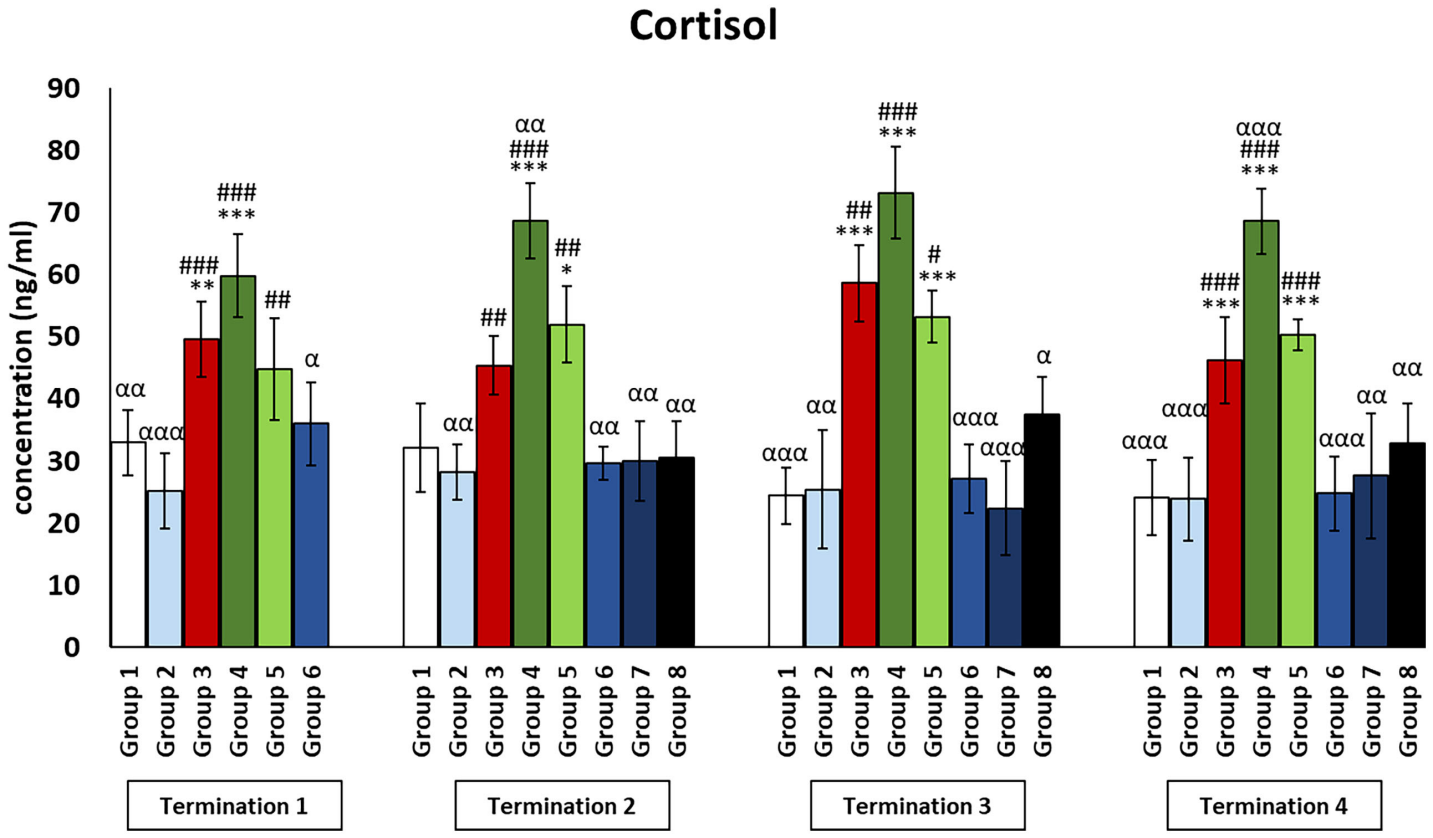
Changes in the levels of cortisol in the blood of chickens receiving phage therapy or antibiotic therapy after 6, 20, 28, and 34 days of experiments. Results are presented as mean values ± SD. Statistical analyses were performed by Kruskal–Wallis test and post-hoc Dunn test or ANOVA and post-hoc Tukey’s test. The significance of differences between controls and particular treated groups is observed and marked by the following: asterisks (*) vs. saline control; (#) vs. phage control (group 2); (a) vs. infected control (group 3); (g) vs. termination 2. p < 0.001 (***, ###, ααα), p < 0.01 (**, ##, αα), and p < 0.05 (*, #, α).

Cortisol levels in all groups treated with a phage cocktail (groups 6–8) were not significantly different from those in the non-infected control groups (groups 1 and 2) throughout the experiment (**Figure 9**). Interestingly, cortisol levels in the enrofloxacin-treated group (group 4) were dramatically increased compared to those in the uninfected control groups (groups 1 and 2) and even higher than those in the *Salmonella*-infected control group (group 3) (termination 2, *p* ≤ 0.01; termination 4; *p* ≤ 0.001). Next, cortisol levels in the colistin-treated group (group 5) were not different from those in the saline-treated control group (group 1) on day 6 (termination 1) but were increased on day 20 (termination 2, *p* ≤ 0.05), day 28 (termination 3, *p* ≤ 0.001), and day 34 (termination 4, *p* ≤ 0.001) of the experiment.

## Discussion

Since phage therapy is generally considered a promising alternative to the treatment of infected animals with antibiotics, and infections with *S.* Typhimurium belong to the most important problems in the aviculture of chickens, our current work is focused on the comparison of effects of phage therapy and treatment with antibiotics in the model of *S.* Typhimurium-infected chickens (11–13). In our recent work, we have demonstrated that the phage therapy with a cocktail of two phages, vB_SenM-2 and vB_Sen-TO17, is as effective in the eradication of *S.* Typhimurium from the chicken gut as the use of enrofloxacin or colistin, which are antibiotics employed routinely in veterinary medicine (14). When either phage therapy or antibiotic therapy started 1 day after the bacterial infection of chickens, the elimination of *S.* Typhimurium from feces and cloaca swabs was complete the next day after the onset of the treatment. However, the delayed onset of phage therapy prolonged the presence of *S.* Typhimurium cells in these biological materials for up to 2–4 days (14). Interestingly, in the above-discussed recent study, no appearance of *S.* Typhimurium cells resistant to either phages or antibiotics used in the experiment could be detected (14). An important observation was that orally administered phages could be found in various organs of chickens, including the kidneys, muscles, spleen, liver, heart, and brain (14). This raised a question about the putative influence of phage therapy on the physiology of the birds. Since the primary reaction of the bird or mammalian body to the presence of alien macromolecules is the immune response, it was important to test such a response in the experimental system described above.

Considering the unanswered question presented above, the major aim of this study was to analyze the immune response to the bacteriophages (vB_SenM-2 and vB_Sen-TO17) and commonly used antibiotics (enrofloxacin and colistin) in controlling *S. enterica* serovar Typhimurium infection in chickens. To our knowledge, this is the first time the effect of bacteriophage administration and the above-mentioned antibiotics on a large battery of parameters of the immune system has been investigated. Moreover, this study may contribute to the widespread use of phages in the non-invasive control of bacterial infections in poultry.

The experimental infection model used in the present experiments had specific kinetic parameters. Clinical signs, observations of the feathers, the degree of redness and discoloration of the comb, as well as the inflammatory markers tested, and the histological evaluation of the internal organs taken confirmed that the infection was chronic and latent, with no visible external symptoms. Comparison of the effects of different doses of two bacterial strains, *Salmonella* Galinarum and *S*. Typhimurium, showed that in the first case, chicken mortality significantly increased even with the application of 10^6^ CFU/ml, while inoculation with a dose of 10^9^ CFU/ml of *S*. Typhimurium did not result in the appearance of any external, clinical signs and did not affect chicken mortality (18). Feces were collected daily at a fixed time, using medical pads that were placed in boxes that housed only chickens from the particular experimental group. Moreover, the swabs from walls, doors, floors of the boxes, and water and feed samples were collected and analyzed for the presence of *S. enterica* to demonstrate no such contamination. Regarding the kinetics of infection development, both our observations and literature data confirm that in the case of *S.* Typhimurium, the highest number of bacteria was detected in cecal contents, and low or below detection threshold in the liver and spleen, and did not change over the course of the experiment (18). The pathophysiological changes resulting from the chronic nature of the infection were manifested at a later stage primarily by a decrease in body weight (not all chickens) and an abnormal histopathological picture of internal organs. First of all, tissue damage and inflammatory changes were evident, resulting from infiltration of heterophils and lymphocytes in the liver and kidneys and, to a lesser extent, in the spleen (data not shown). As a result of oral supplementation with the phage cocktail, in addition to the elimination of bacterial cells, an increase in the body weight of chickens was observed, which, however, did not exceed the threshold of statistical significance, as well as the absence of changes in the histopathological picture of the organs examined. In contrast, the opposite effects were observed after antibiotic therapy, especially after enrofloxacin administration, which negatively affected the behavior of chickens (episodes of aggression and auto-aggression) and the appearance and structure of feathers and, above all, generated a number of pathological changes visible in tissues and organs, including the brain (data not shown).

Huang etal. (19) examined IL-6 and IFN-γ serum concentrations after a single administration of CKT1 phage to *Salmonella pullorum*-infected chickens. The analysis included two time points: 3 and 6 days after bacteria detection. IFN-γ levels were virtually undetectable, while IL-6 levels were significantly elevated on day 6 after infection, which was interpreted by the authors as a high efficiency of the phage in eliminating bacterial cells. In contrast, our analyses showed that as early as day 4 after infection, the development of the inflammatory response was so vigorous that the phage therapy had no inhibitory potential. Because Huang et al. (19) presented only the concentration of a single cytokine and did not include the level of cytokines that reveal anti-inflammatory effects, indicating that a compensatory mechanism works properly, the evaluation of the efficacy of the phage used could not be performed precisely. In contrast, Xue et al. (20) analyzed pro-inflammatory parameters in organs collected from mice supplemented with phage X1, 6 h after infection with the pathogenic bacteria *Yersinia enterocolitica.* This bacterium causes the third most common severe zoonosis, which is a huge problem, especially in European Union countries. Similar to our findings, the levels of pro-inflammatory cytokines, IL-6, TNF-α, and IL-1β, tested after bacteriophage administration, were significantly lower than in the group with developed bacterial infection, treated with PBS alone. The observed effects lasted up to 72 h and confirmed the safety and efficacy of phage therapy against problematic bacterial infections. Our results also clearly show that the phage therapy used does not disturb the balance between pro-inflammatory cytokines and those exerting anti-inflammatory actions. The situation is different with the antibiotics used. This is particularly evident with regard to enrofloxacin, which causes a non-physiological decrease in pro-inflammatory cytokines, as well as inhibits the potential of cytokines that exert anti-inflammatory actions, as expressed by reduced levels of IL-10 and IL-4. Only a few available literature data indicate that antibiotics of the quinolone group, to which enrofloxacin belongs, cause the deregulation of mRNA levels of genes coding for some cytokines, like IL-1α, TNF-α, IL-2, IL-3, or IL-4. It was suggested that this type of disruption of cytokine synthesis, which is crucial for the maintenance of immune homeostasis, resembles the activation of the bacterial SOS system. Moreover, in mammalian cells, this situation reflects the organism’s response to severe stress or DNA damage (21). Enrofloxacin is an old fluoroquinolone that has been used in veterinary medicine since 1991. When the phrase “enrofloxacin immune system chicken” is used as a query in the PUBMED database, only 22 records appear. All these studies have led us to agree with the statement that enrofloxacin can cause changes in the immune system, including the chicken immune system. However, knowledge regarding the effects of enrofloxacin on protein levels and the number of cells in the immune system of hens is insufficient, so we decided to comprehensively investigate this issue. Although this antibiotic has a broad spectrum of action, covering both many Gram-negative and Gram-positive bacterial species, it has many side effects. A recent article summarized data on both the efficacy of enrofloxacin and many side effects on the skeletal, nervous, and immune systems. That paper examined how enrofloxacin affected the immune systems of cattle, pigs, carp, and striped bass (16). Qiu et al. (22) demonstrated that exposure of carp macrophages to enrofloxacin caused activation of the NF-κB pathway and induction of an NF-κB-based immune response, which included the formation of reactive oxygen species and expression of cytokines. In turn, Strzępa et al. (23) showed that the levels of IL-4, IL-10, IL-5, and IL-13 were reduced significantly in mice treated with enrofloxacin, prior to immunization with ovalbumin. Sun et al. (24) showed that the administration of enrofloxacin for 3 weeks significantly disrupts gut microbiota and affects the expression of mRNAs coding for inflammatory mediators in the mouse colon. Indeed, the increased expression was found primarily in genes encoding pro-inflammatory cytokines, like IFN-γ, TNF-α, IL-1β, and IL-6. In addition, an analogous trend was observed for the cytokines that exert anti-inflammatory actions, exemplified by IL-10, as well as the Th17 lymphocyte effector cytokines, IL-17 and IL-23. Moreover, enrofloxacin treatment resulted in an elevated IFN-γ to IL-4 ratio, suggesting an enhanced response mediated by Th1 rather than Th2 lymphocytes. In turn, Kowalczyk et al. (25) evaluated the effect of perinatal enrofloxacin administration on the immune system in adult offspring mice. They showed that long-term administration of this antibiotic disrupts the T lymphocyte-dependent immune response and increases the risk of skin allergies in adult individuals. The hypothesis that enrofloxacin should not always be the first antibiotic of choice for the prevention and treatment of bacterial infections in chickens was also confirmed by Ma et al. (26). Interestingly, they showed that in the groups treated with different doses of enrofloxacin, the number of bacterial cells is significantly higher than in the untreated groups and persists for a longer period of time. Moreover, this dose-dependent trend applies not only to the intestines but also to other internal organs. In contrast, the effects of colistin on cytokine balance were not well studied and described. Wang et al. (27) showed an immunomodulatory effect of colistin on rat macrophages. The observed impact was dependent on the activation of the p38/MAPK pathway, as well as on the dose used. The lowest dose of this antibiotic (5 µg/ml) resulted in a significant increase in the concentration of some cytokines, TNF-α, IL-1β, and IL-6. Furthermore, Bauquier et al. (28) analyzed the effect of polymyxin B (belonging to the same group of antibiotics) on TNF-α levels in blood collected from horses that had previously been incubated with three different types of bacterial toxins. The results showed that the anti-inflammatory activity of colistin was only confirmed with lipoteichoic acid, but not with peptidoglycan or lipopolysaccharide. Adverse effects of colistin, mainly in the form of increased oxidative stress parameters, have also been confirmed in studies with rodents. Çelik et al. (29) showed that colistin caused an increase in the levels of the apoptotic and inflammatory markers, like cysteine aspartate-specific protease-3, p53, B-cell lymphoma-2, NF-κB, Bcl-2 associated X protein, TNF-α, and neuronal nitric oxide synthase (nNOS). In addition to the fact that the phage cocktail effectively combated *S. enterica* serovar Typhimurium infection without disrupting the cytokine balance, it significantly increased the concentration of the key cytokine that exerts anti-inflammatory action, IL-10. Even a less than twofold increase in its level is crucial for the functioning of the organism in the face of infection. The levels of interleukins IL-4 and IL-10 in the groups that were infected with *Salmonella* and treated with bacteriophages (groups 6 and 7) did not differ from those of the saline-treated control group (group 1), while they differed significantly from those of the bacteriophage-infected control group (group 3). The interleukins selected by us, IL-4 and IL-10, are examples of those exerting anti-inflammatory actions, which confirm that bacteriophages can have immunomodulatory and anti-inflammatory effects. The results obtained are compatible with the work presented by other teams. To date, it has been shown that bacteriophages were capable of significantly reducing C-reactive protein (CRP) protein levels in humans (5). Moreover, Miernikiewicz et al. (30) showed that the gp12 protein of bacteriophage T4 is able to antagonize the pro-inflammatory effects of lipopolysaccharide (LPS), causing a reduction in IL-1 and IL-6 levels. In addition, it was also confirmed that phages can reduce the production of reactive oxygen species (ROS) and activation of nuclear factor NF-κB while inducing IL-10 and IL-1 antagonists (31). IL-10 is a cytokine with a strong immunosuppressive effect, which determines the maintenance of peripheral immune tolerance and protects tissues from damage resulting from severe inflammation. This potential has been confirmed, among other things, through experiments on IL-10-deficient mice, which had a high mortality rate due to an increased response to LPS or different pathogens (32). Another important aspect of phage’s potential to combat the negative effects of bacterial infection is to stimulate the release of IL-4. It exhibits broad pleiotropic effects not only by modulating the activity of T and B lymphocytes but also by affecting different classes of cells, regulating both innate and adaptive immunity. In addition, IL-4 affects the function of neutrophils, which are the first line of defense in fighting bacterial infections. Although this relationship is not yet fully explored, it is postulated that in the course of infection, when neutrophils support macrophages and their numbers increase significantly, Th2 lymphocytes release IL-4 to inhibit their activity, thus limiting the occurrence of potential tissue damage, resulting from excessive neutrophil activation (33). The analyzed cytokines that exert a range of anti-inflammatory activities are also important in the control of bacterial infections because they stimulate the transformation of macrophages into one of the two key phenotypes, M1 or M2. Macrophages are a part of the innate immune response, which determines the recognition, phagocytosis, and elimination of bacteria through the release of reactive oxygen species or proteolytic enzymes and other agents. Differentiation into one of the aforementioned macrophage phenotypes is mainly determined by exposure to a specific type of cytokine. Th1 cytokines (IFN-γ and TNF-α) cause polarization into M1 macrophages, while Th2-specific cytokines (IL-4, IL-10, and IL-13) promote M2 macrophages (34). Macrophages classified as M1 phenotype stimulate the pro-inflammatory phase of the immune response, while M2 macrophages are responsible for anti-inflammatory activities to rapidly eliminate the bacterial infection and recover the organism (35).

An important aspect that must be taken into account is the immune status of the organism prior to therapy. This relationship is evident not only in animal models but also in clinical studies. Reports in the literature indicate that some bacteriophages may interact in a manner analogous to cytokine production after lipopolysaccharide stimulation, which is an immunologically active component of the cell wall structure of Gram-negative bacteria (36). An interesting relationship was observed for the pro-inflammatory cytokine, TNF-α. Patients who had low-to-moderate serum levels of TNF-α at baseline showed an increase in this parameter after phage therapy. In addition, bacteriophages can stimulate IFN release by T lymphocytes through interaction with antigen-presenting dendritic cells (37). In our study, administration of the phage cocktail alone did not alter the plasma levels of the tested pro-inflammatory cytokines.

Another important aspect is the potential effect of phage cocktails on immunocompetent cells. Numerous studies confirmed that phages exhibit both prophylactic and anti-inflammatory potential in overcoming various bacterial infections, manifested by reducing the secretion of cytokines and chemokines, such as IL-12, IL-13, CCL5, or hematopoietic factor granulocyte colony-stimulating factor (G-CSF), by immune cells. Despite the observed anti-inflammatory activity, there were no changes in the numbers of immunocompetent cells: lymphocytes, monocytes, or neutrophils in the serum. An analogous situation was observed with respect to significant changes in the number of macrophages, T and B lymphocytes, and dendritic cells in peripheral organs: spleen, liver, and lymph nodes (38); (39). Our results also confirm that administration of bacteriophages immediately or 2 days after detection of bacterial infection did not significantly affect the number of peripheral blood T lymphocytes, which did not differ from that observed in the control group. The opposite effect was observed for antibiotic therapy, especially with enrofloxacin. Its use resulted in a significant decrease in the percentage of B lymphocytes, as well as T-lymphocyte subpopulations: Th (CD4+) and Tc (CD8+). Riesbeck et al. (40) reported that the administration of enrofloxacin especially during the first few days of chicken life can negatively affect the immune response by disrupting the ratio between lymphocyte subpopulations. This pathological state may persist even throughout the rearing period. In addition, they observed that enrofloxacin attenuates humoral immunity, expressed by antibody production, while it stimulates cellular immunity, which involves T lymphocytes as major effector cells. Colistin, however, reduces lymphoid follicle proliferation, which also results in a decrease in the percentage of circulating lymphocytes, especially B lymphocytes (41). Interestingly, it is speculated that the mentioned IL-10 is a key molecule in the context of *S. enterica* infection, due to the fact that it allows masking its presence, thus avoiding overactivation of the host immune system. It stimulates the proliferation of T_reg_ cells, thereby balancing the immune response to the ongoing infection, thus limiting the potential tissue damage. Salazar et al. (42) showed that during the infection of *S. enterica* serovar Typhimurium, B lymphocytes provide an important additional signal to T lymphocytes that promote the formation of T_reg_ cells. It is noteworthy that the bacterium is able to infect and reside latently inside B lymphocytes, in their precursor cells in the bone marrow, or in plasma cells, which is another factor that stimulates the differentiation of T lymphocytes into T_reg_ cells. Therefore, the ratio of T and B lymphocytes is crucial for the course of *S. enterica* serovar Typhimurium infection.

An even less frequently analyzed parameter was the effect of administered bacteriophages on stress hormones. In our study, we focused on corticosterone, which level is 100 times higher than that of cortisol in commercial birds’ blood (43). Elevated corticosterone levels result in a number of changes when it comes to immune system parameters. Among other things, there is a modification in the ratio of heterophils to lymphocytes, in which the number decreases. In addition, parameters such as the size of the cells, shape, or granulation change. The mRNA expression of pro-inflammatory cytokines and chemokines also increases, and lymphocyte proliferation is suppressed. Sustained exposure to stressors disrupts the mechanisms designed to restore homeostasis, which translates into weakened immunity and has negative consequences for the health of the entire organism (44). In our study, corticosterone levels were significantly elevated in the group with a bacterial infection. Interestingly, a similar situation was also noted in the groups after antibiotic therapy. However, administration of the phage cocktail did not cause an increase in the concentration of the described hormone. One of the few reports on the relationship between phage therapy and stress axis activity involved fish. The increase in cortisol levels in response to an acute stressor is usually short-lived and quickly normalizes. It is presumed to be the result of bacterial LPS stimulation rather than the applied phage therapy (45). Similar results were obtained by Salazar et al. (42), who observed elevated cortisol levels in the European eel (*Anguilla anguilla*) blood within 24 h after administration of phage preparation, BAFADOR. Within a week, this parameter normalized, and its concentration did not differ significantly from that in the control group.

It is worth to note that our work was not the first study on the use of phage therapy for the treatment of *S. enterica*-infected chickens. The first paper in this field has been published in 1991 (46), and then over 100 reports described trials focused on the use of bacteriophages to treat such infections, as summarized recently (47). The common conclusion arising from those studies was that the phage therapy indicated efficacy in the elimination of *Salmonella* cells and the normalization of the gut microbiome (19, 48, 49). However, the novelty of this work was to compare the effects of phage therapy and antibiotic therapy on the immune response of chickens. This was important, as, despite the hope that the use of bacteriophages might replace antibiotic therapy in the future, there are still concerns about the efficacy and safety of the phage therapy. The potential drawbacks include, but are not limited to, the possibility of selection of phage-resistant bacteria, problems with efficient delivery of phages to various organs, putative toxic effects of relatively large amounts of compounds released into the host body after sudden disruption of bacterial cells by phages, and possible effects of phages on the gut microbiome and modulation of the immune response, as reviewed and discussed recently (50). Indeed, bacteriophages have even been proposed as being pathogens of animals and humans (51–53). Moreover, there are serious problems with the legal aspects of the use of bacteriophages as “drugs” (54). In light of these doubts, our recent work (14) and this study provided important evidence that in the treatment of *S.* Typhimurium-infected chickens, phage therapy is effective in the elimination of the pathogenic bacteria and relatively safe, at least in the aspect of the immune response of animals.

In conclusion, having analyzed the immune response of chickens to the administration of the phage cocktail, we conclude that in non-infected as well as *S. enterica* serovar Typhimurium-infected animals, it does not cause adverse effects that would negatively affect the cytokine balance, immune cell subpopulation, or other key parameters of the immune system.

## Data availability statement

The original contributions presented in the study are included in the article/**Supplementary Material**. Further inquiries can be directed to the corresponding author.

## Ethics statement

This study was reviewed and approved by The Local Ethics Committee for Experimental Animals in Olsztyn (permission number 62/2019 dated July 30, 2019).

## Author contributions

ŁG prepared the phage cocktail, participated in sections of terminated chickens, prepared blood for future experiments, performed the analysis of levels of cytokines and stress hormones in chicken blood plasma, performed the analysis of levels of blood morphological parameters, analyzed the percentage of lymphocytes in peripheral blood, performed the statistical analysis, co-drafted the manuscript, and prepared the visualization of the results. GW participated in sections of terminated chickens and participated in analyses of results and co-drafted the manuscript. AW participated in sections of terminated chickens, participated in the analysis of levels of cytokines, was the principal investigator of the project, analyzed data, and co-drafted the manuscript. MP presented the concept of the study, planned and coordinate experiments, participated and coordinated the sections of terminated chickens, participated in the analysis of levels of cytokines and stress hormones in chicken blood plasma, participated in the analysis of levels of blood morphological parameters and participated in the analysis of the percentage of lymphocytes in peripheral blood, and co-drafted the manuscript. All authors contributed to the article and approved the submitted version.

## Funding

This work was supported by the National Science Centre (Poland) within project grant no. 2017/27/B/NZ9/00393.

## Acknowledgments

The authors thank the research team of the Pavilion of Experimental Birds Infections, University of Warmia and Mazury, Olsztyn, Poland, for their services during experiments with chickens. They also thank Joanna Morcinek, MSc, for her technical assistance in preparing **Figure 1**.

## Conflict of interest

The authors declare that the research was conducted in the absence of any commercial or financial relationships that could be construed as a potential conflict of interest.

## Publisher’s note

All claims expressed in this article are solely those of the authors and do not necessarily represent those of their affiliated organizations, or those of the publisher, the editors and the reviewers. Any product that may be evaluated in this article, or claim that may be made by its manufacturer, is not guaranteed or endorsed by the publisher.

## Supplementary material

Supplementary Figure 1 Changes in the levels of IL-6 in the blood of chickens receiving phage therapy or antibiotic therapy after 6, 20, 28 and 34 days of experiments. Results are presented as mean values ± SD. Statistical analyses were performed by Kruskal-Wallis test and *post-hoc* Dunn test or ANOVA and *post-hoc* Tukey test. The significance of differences between controls and particular treated groups are observed and marked by: asterisks (*) vs. saline control; (#) vs. phage control (group 2); (α) vs. infected control (group 3); (γ) vs. termination 2. p < 0.001 (***, ###, ααα, γγγ); p< 0.01 (**, ##, αα, γγ); p< 0.05 (*, #, α, γ).

Supplementary Figure 2 Changes in the levels of IL-8 in the blood of chickens receiving phage therapy or antibiotic therapy after 6, 20, 28 and 34 days of experiments. Results are presented as mean values ± SD. Statistical analyses were performed by Kruskal-Wallis test and *post-hoc* Dunn test or ANOVA and *post-hoc* Tukey test. The significance of differences between controls and particular treated groups are observed and marked by: asterisks (*) vs. saline control; (#) vs. phage control (group 2); (α) vs. infected control (group 3); (γ) vs. termination 2. p < 0.001 (***, ###, ααα, γγγ); p< 0.01 (**, ##, αα, γγ); p< 0.05 (*, #, α, γ).

Supplementary Figure 3 Changes in the levels of IL-12 in the blood of chickens receiving phage therapy or antibiotic therapy after 6, 20, 28 and 34 days of experiments. Results are presented as mean values ± SD. Statistical analyses were performed by Kruskal-Wallis test and *post-hoc* Dunn test or ANOVA and *post-hoc* Tukey test. The significance of differences between controls and particular treated groups are observed and marked by: asterisks (*) vs. saline control; (#) vs. phage control (group 2); (α) vs. infected control (group 3); (γ) vs. termination 2. p < 0.001 (***, ###, ααα, γγγ); p< 0.01 (**, ##, αα, γγ); p< 0.05 (*, #, α, γ).

Supplementary Figure 4 Changes in lymphocyte Th CD4+ counts in the blood of chickens receiving phage therapy or antibiotic therapy after 6, 20, 28 and 34 days of experiments. Results are presented as mean values ± SD. Statistical analyses were performed by Kruskal-Wallis test and *post-hoc* Dunn test or ANOVA and *post-hoc* Tukey test. The significance of differences between controls and particular treated groups are observed and marked by: asterisks (*) vs. saline control; (#) vs. phage control (group 2); (α) vs. infected control (group 3); (γ) vs. termination 2. p < 0.001 (***, ###, ααα, γγγ); p< 0.01 (**, ##, αα, γγ); p< 0.05 (*, #, α, γ).

Supplementary Figure 5 Changes in lymphocyte Tc CD8+ counts in the blood of chickens receiving phage therapy or antibiotic therapy after 6, 20, 28 and 34 days of experiments. Results are presented as mean values ± SD. Statistical analyses were performed by Kruskal-Wallis test and *post-hoc* Dunn test or ANOVA and *post-hoc* Tukey test. The significance of differences between controls and particular treated groups are observed and marked by: asterisks (*) vs. saline control; (#) vs. phage control (group 2); (α) vs. infected control (group 3); (γ) vs. termination 2. p < 0.001 (***, ###, ααα, γγγ); p< 0.01 (**, ##, αα, γγ); p< 0.05 (*, #, α, γ).

Supplementary Figure 6 Changes in the absolute leukocyte counts in the blood of chickens receiving phage therapy or antibiotic therapy after 6, 20, 28 and 34 days of experiments. Results are presented as mean values ± SD. Statistical analyses were performed by Kruskal-Wallis test and *post-hoc* Dunn test or ANOVA and *post-hoc* Tukey test. The significance of differences between controls and particular treated groups are observed and marked by: asterisks (*) vs. saline control; (#) vs. phage control (group 2); (α) vs. infected control (group 3); (γ) *vs*. termination 2. p < 0.001 (***, ###, ααα, γγγ); p< 0.01 (**, ##, αα, γγ); p<
