# Supplementary material for: Highly different effects of phage therapy and antibiotic therapy on immunological responses of chickens infected with Salmonella enterica serovar Typhimurium
Source: Front Immunol. 2022 Sep 23;13:956833. doi: 10.3389/fimmu.2022.956833 (PMC9539762; doi:10.3389/fimmu.2022.956833)
Supplement: Supplementary Figure 1 — Changes in the levels of IL-6 in the blood of chickens receiving phage therapy or antibiotic therapy after 6, 20, 28 and 34 days of experiments. Results are presented as mean values ± SD. Statistical analyses were performed by Kruskal-Wallis test and post-hoc Dunn test or ANOVA and post-hoc Tukey test. The significance of differences between controls and particular treated groups are observed and marked by: asterisks (*) vs. saline control; (#) vs. phage control (group 2); (α) vs. infected control (group 3); (γ) vs. termination 2. p < 0.001 (***, ###, ααα, γγγ); p< 0.01 (**, ##, αα, γγ); p< 0.05 (*, #, α, γ). [file DataSheet_1.docx]

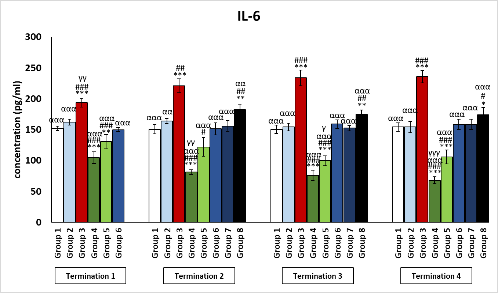
Supplementary Figure 1

Changes in the levels of IL-6 in the blood of chickens receiving phage therapy or antibiotic therapy after 6, 20, 28 and 34 days of experiments. Results are presented as mean values ± SD. Statistical analyses were performed by Kruskal-Wallis test and post-hoc Dunn test or ANOVA and post-hoc Tukey test. The significance of differences between controls and particular treated groups are observed and marked by: asterisks (*) vs. saline control; (#) vs. phage control (group 2); (α) vs. infected control (group 3); (γ) vs. termination 2. p < 0.001 (***, ###, ααα, γγγ ); p< 0.01 (**, ##, αα, γγ); p< 0.05 (*, #, α, γ).


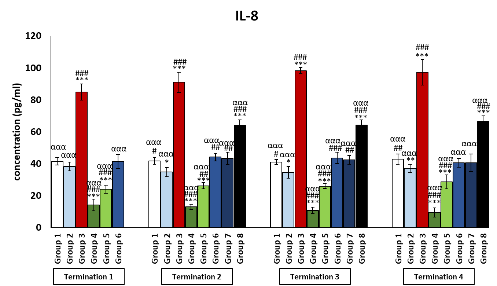


Supplementary Figure 2

Changes in the levels of IL-8 in the blood of chickens receiving phage therapy or antibiotic therapy after 6, 20, 28 and 34 days of experiments. Results are presented as mean values ± SD. Statistical analyses were performed by Kruskal-Wallis test and post-hoc Dunn test or ANOVA and post-hoc Tukey test. The significance of differences between controls and particular treated groups are observed and marked by: asterisks (*) vs. saline control; (#) vs. phage control (group 2); (α) vs. infected control (group 3); (γ) vs. termination 2. p < 0.001 (***, ###, ααα, γγγ ); p< 0.01 (**, ##, αα, γγ); p< 0.05 (*, #, α, γ).


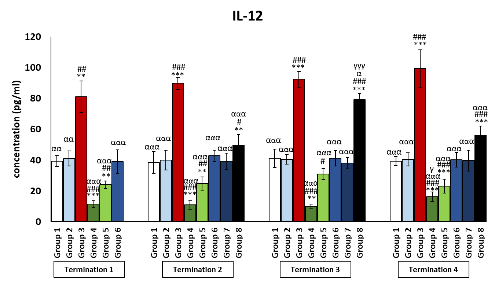


Supplementary Figure 3

Changes in the levels of IL-12 in the blood of chickens receiving phage therapy or antibiotic therapy after 6, 20, 28 and 34 days of experiments. Results are presented as mean values ± SD. Statistical analyses were performed by Kruskal-Wallis test and post-hoc Dunn test or ANOVA and post-hoc Tukey test. The significance of differences between controls and particular treated groups are observed and marked by: asterisks (*) vs. saline control; (#) vs. phage control (group 2); (α) vs. infected control (group 3); (γ) vs. termination 2. p < 0.001 (***, ###, ααα, γγγ ); p< 0.01 (**, ##, αα, γγ); p< 0.05 (*, #, α, γ).


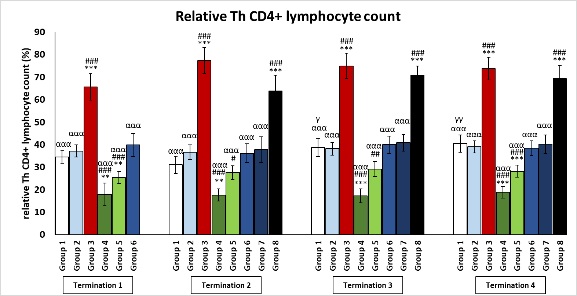


Supplementary Figure 4

Changes in lymphocyte Th CD4+ counts in the blood of chickens receiving phage therapy or antibiotic therapy after 6, 20, 28 and 34 days of experiments. Results are presented as mean values ± SD. Statistical analyses were performed by Kruskal-Wallis test and post-hoc Dunn test or ANOVA and post-hoc Tukey test. The significance of differences between controls and particular treated groups are observed and marked by: asterisks (*) vs. saline control; (#) vs. phage control (group 2); (α) vs. infected control (group 3); (γ) vs. termination 2. p < 0.001 (***, ###, ααα, γγγ ); p< 0.01 (**, ##, αα, γγ); p< 0.05 (*, #, α, γ).


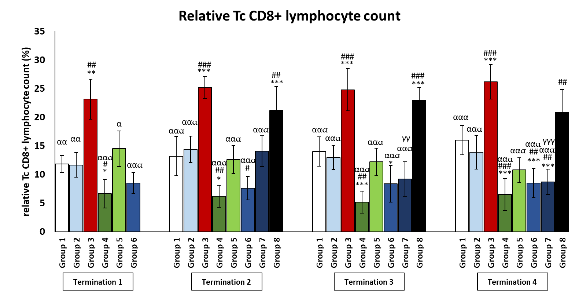


Supplementary Figure 5

Changes in lymphocyte Tc CD8+ counts in the blood of chickens receiving phage therapy or antibiotic therapy after 6, 20, 28 and 34 days of experiments. Results are presented as mean values ± SD. Statistical analyses were performed by Kruskal-Wallis test and post-hoc Dunn test or ANOVA and post-hoc Tukey test. The significance of differences between controls and particular treated groups are observed and marked by: asterisks (*) vs. saline control; (#) vs. phage control (group 2); (α) vs. infected control (group 3); (γ) vs. termination 2. p < 0.001 (***, ###, ααα, γγγ ); p< 0.01 (**, ##, αα, γγ); p< 0.05 (*, #, α, γ).


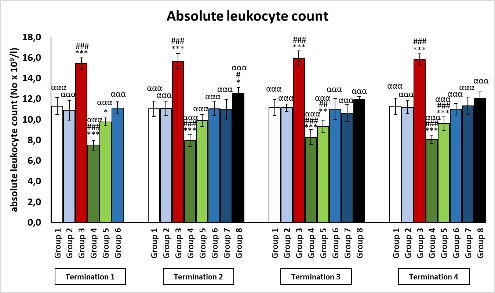


Supplementary Figure 6

Changes in the absolute leukocyte counts in the blood of chickens receiving phage therapy or antibiotic therapy after 6, 20, 28 and 34 days of experiments. Results are presented as mean values ± SD. Statistical analyses were performed by Kruskal-Wallis test and post-hoc Dunn test or ANOVA and post-hoc Tukey test. The significance of differences between controls and particular treated groups are observed and marked by: asterisks (*) vs. saline control; (#) vs. phage control (group 2); (α) vs. infected control (group 3); (γ) vs. termination 2. p < 0.001 (***, ###, ααα, γγγ ); p< 0.01 (**, ##, αα, γγ); p< 0.05 (*, #, α, γ).
